# Mortality and Clinical Outcomes among Patients with COVID-19 and Diabetes

**DOI:** 10.3390/medsci9040065

**Published:** 2021-10-26

**Authors:** Viny Kantroo, Manjit S. Kanwar, Piyush Goyal, Deepak Rosha, Nikhil Modi, Avdhesh Bansal, Athar Parvez Ansari, Subhash Kumar Wangnoo, Sanjay Sobti, Sudha Kansal, Rajesh Chawla, Sanjiv Jasuja, Ishan Gupta

**Affiliations:** 1 Department of Respiratory, Critical Care and Sleep Medicine, Indraprastha Apollo Hospitals, New Delhi 110076, India; kanwarms53@gmail.com (M.S.K.); piyushgoyal1993@yahoo.com (P.G.); drosha@hotmail.com (D.R.); Nikmodi2309@gmail.com (N.M.); Avdheshb@gmail.com (A.B.); atharparvez@yahoo.co.uk (A.P.A.); Sanjay_Sobti@apollohospitalsdelhi.com (S.S.); kansalsudha08@gmail.com (S.K.); drchawla@hotmail.com (R.C.); dr.ishangupta90@gmail.com (I.G.); 2 Department of Apollo Centre of Diabetes and Endocrinology, Indraprastha Apollo Hospitals, New Delhi 110076, India; subhashwang@hotmail.com; 3 Department of Nephrology and Kidney Transplant, Indraprastha Apollo Hospitals, New Delhi 110076, India; sanjivjasuja@yahoo.com

**Keywords:** COVID-19, diabetes mellitus, hypertension, mortality rate, coronary artery disease, chronic kidney disease

## Abstract

***Background*** Diabetes mellitus (DM) is a decisive risk factor for severe illness in coronavirus disease 2019 (COVID-19). India is home to a large number of people with DM, and many of them were infected with COVID-19. It is critical to understand the impact of DM on mortality and other clinical outcomes of COVID-19 infection from this region. ***Aims*** The primary objective of our study was to analyze the mortality rate in people with DM infected with COVID-19. The secondary objectives were to assess the effect of various comorbidities on mortality and study the impact of DM on other clinical outcomes. ***Methods*** This is a retrospective study of COVID-19 infected patients admitted to a tertiary care hospital in north India in the early phase of the pandemic. ***Results*** Of the 1211 cases admitted, 19 were excluded because of incomplete data, and 1192 cases were finally considered for analysis. DM constituted 26.8% of total patients. The overall mortality rate was 6.1%, and the rate was 10.7% in the presence of diabetes (*p* < 0.01, OR 2.55). In univariate analysis, increased age, chronic kidney disease (CKD), coronary artery disease (CAD), stroke, and cancer were associated with mortality. On multiple logistic regression, the independent predictors of mortality were CAD, CKD, and cancer. Breathlessness and low SpO_2_ at presentation, extensive involvement in CXR, and elevated ANC/ALC ratio were also significantly associated with mortality. ***Conclusions*** The presence of comorbidities such as DM, hypertension, CAD, CKD, and cancer strongly predict the risk of mortality in COVID-19 infection. Early triaging and aggressive therapy of patients with these comorbidities can optimize clinical outcomes.

## 1. Introduction

Coronavirus disease 2019 (COVID-19), caused by severe acute respiratory syndrome coronavirus 2 (SARS-CoV-2), was declared a global pandemic by the World Health Organization on 11 March 2020 and has affected millions of people across the globe. As reported in the meta-analysis by Shi et al. [1], the mortality rate varied between 3.5% to 61% in different studies. A significant determinant of mortality in COVID-19 is the presence of pre-existing comorbidities [1,2,3,4,5,6]. Among the various comorbidities, diabetes mellitus (DM) has emerged as a critical risk factor for severe disease and mortality in COVID-19 [6,7].

People living with DM are predisposed to develop both bacterial and viral pulmonary disease [8]. The close relationship between DM and infection is well recognized [9]. Infections in DM carry a higher risk for hospitalization [10]. Diabetes does not increase the risk of acquiring the infection, although it does confer a substantial risk of an adverse outcome in COVID-19 infection [11].

There is a higher risk of mortality and severe disease if individuals with DM develop COVID-19 [12,13]. The pathophysiologic link between DM and adverse outcomes in COVID-19 has been closely studied [14]. The possible mechanisms leading to complicated disease could be due to differential expression of angiotensin-converting enzyme 2 (ACE2) in the lungs, and suppression of innate and adaptative immunity in DM [15]. The relationship between variation of the presence of ACE2 in different tissues in various physiological and pathological states and the severity of COVID-19 infection is not fully understood [16,17,18]. It has been hypothesized that a higher expression of ACE2 in the lungs of individuals with DM might increase the susceptibility to viral entry and replication [19]. The dysregulation of the renin-angiotensin-aldosterone system in DM might also modulate the disease course [20,21].

Additionally, DM often coexists with other comorbidities such as obesity, hypertension, cardiovascular disease (CVD), and chronic kidney disease (CKD), which can adversely impact the outcome [11,14,22,23,24]. The low-grade chronic inflammation in DM also has been presumed to increase the susceptibility to a cytokine storm, a critical and fulminant event associated with rapid deterioration during the second week [25]. The specific adaptations in DM that trigger the pro-inflammatory cascade include an increase in the number of CD45+ T cells and alteration in the balance between Th17 cells and Treg cells [26,27].

Individuals with DM are at increased risk of endothelial dysfunction and hypercoagulability, factors known to be associated with adverse outcomes in COVID-19 infection [28]. Endothelial dysfunction is one of the primary mechanisms behind the cardiovascular complications observed in DM [29,30]. Endothelial damage in DM is multifactorial and can result from hyperglycemia, oxidative stress, impaired insulin signaling, altered expression of adhesion molecules, and attenuated production of nitric oxide [31,32,33,34]. A vulnerable endothelium in DM is susceptible to further compromise after COVID-19 infection and can predispose to cardiovascular diseases [19]. The endothelial dysfunction could also contribute to the hypercoagulability that is noted in severe COVID-19 infection [35].

In this study, we have retrospectively analyzed the impact of DM on mortality and other clinical outcomes in 1211 consecutive patients admitted with COVID-19 infection to a tertiary-care hospital. This is one of the largest reported cohorts of patients with COVID-19 infection from southeast Asia. We have additionally studied the relationship between clinical findings at presentation and various comorbidities commonly associated with DM and mortality in this cohort. An understanding of the influence of DM and associated conditions on the outcome of COVID-19 infection is essential for optimizing management and streamlining resources in countries with a large burden of DM.

## 2. Aims and Objectives

Our study is a retrospective evaluation of 1211 consecutive COVID-19 infected patients admitted to a tertiary care hospital in north India. The primary objective was to analyze the mortality rate in COVID-19 and coexisting DM. The secondary objectives were to examine the effects of age, gender, hypertension, coronary artery disease (CAD), CKD, stroke, cancer, and chronic obstructive pulmonary disease (COPD) or bronchial asthma on mortality. We also investigated the influence of DM on other outcomes such as oxygen requirement, length of hospitalization, intensive care unit (ICU) admission, length of ICU stay, and ventilatory requirement.

## 3. Materials and Methods

### 3.1. Study Design and Participants

We did a retrospective analysis of 1211 COVID-19 infected patients in Indraprastha Apollo Hospitals, Sarita Vihar, New Delhi, India, a 710 bedded tertiary-care hospital. Since this is a retrospective collection of data, the consent waiver was obtained from the biomedical ethics committee as per the hospital protocols. The medical records of consecutive patients admitted between 25 March 2020 and 3 August 2020 were analyzed. All patient data were anonymized. The diagnosis of COVID-19 infection was confirmed based on a positive reverse transcription-polymerase chain reaction (RT-PCR) test. Patients with strong clinical and/or radiological suspicion but with a negative RT-PCR test underwent a second RT-PCR. If the second RT-PCR report was negative, the patients were omitted.

### 3.2. Data Collection

We retrieved health records of consecutive patients belonging to all age groups admitted with COVID-19 infection from the hospital medical records system. Demographic information (age and gender) and underlying comorbidities such as DM, hypertension, CAD, CKD, stroke, cancer, and COPD or bronchial asthma were documented. DM was diagnosed if self-reported or if the glycated hemoglobin (HbA1c) during admission was 6.5% or more. Diagnosis of hypertension, CAD, CKD, stroke, cancer, and COPD or bronchial asthma were either self-reported or confirmed based on past medical records.

The presenting clinical features that were noted included fever, cough, shortness of breath, and headache.

The peripheral oxygen saturation (SpO_2_), chest X-ray (CXR), total leucocyte count (TLC), and the absolute neutrophil count (ANC)/absolute lymphocyte count (ALC) ratio at presentation were recorded. The CXR at presentation was graded by simplifying the criteria proposed by Warren et al. [36]. No involvement on CXR was considered 0, less than 25% involvement was assigned a score of 1, and more than 25% involvement was given a score of 2. All the collected data was reexamined by a different team of doctors.

### 3.3. Study Outcomes

The outcome parameters assessed were mortality, length of hospital stay, oxygen requirement, ICU admission, length of ICU stay, use of a ventilator (non-invasive and invasive), and days on the ventilator.

### 3.4. Statistical Analysis

The IBM SPSS statistics software version 22.0 (IBM Corp, Armonk, NY, USA) was used for analyzing the results. The continuous variables were tested for the normality assumption using the Shapiro–Wilk test. All variables were found to be skewed and were summarized using median and interquartile range (IQR). The categorical variables were presented as counts and percentages. The association between demographic and clinical parameters with the presence of DM was assessed using the Chi-square test for categorical variables and the Mann–Whitney U test for continuous variables. Univariate logistic regression analysis was carried out to determine the risk factors for mortality. All significant factors in the univariate analysis were included for multivariate logistic regression analysis. The *p*-value was considered significant at a 5% level of significance for all the analyses.

## 4. Results

### 4.1. Demographic Features

A total of 1211 cases were diagnosed to have COVID-19 during the study period. Nineteen patients were excluded from the analysis because of incomplete data. The number of patients finally included in the study was 1192, and DM was present in 319 (26.8%). The baseline characteristics of the patients are summarized in Table 1.

**Figure 1:**
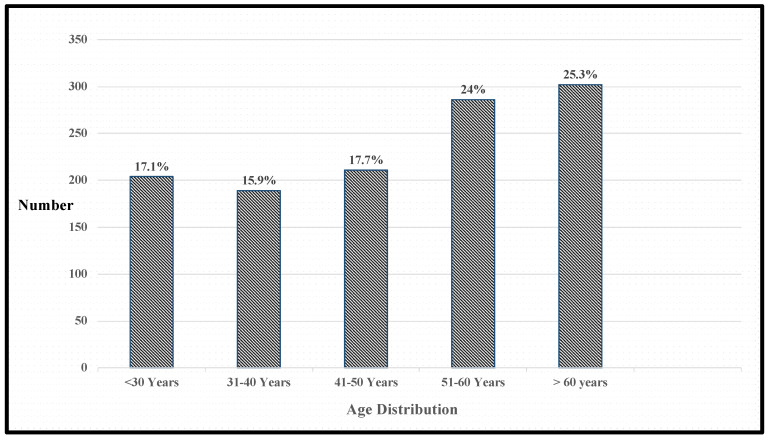
Age-wise distribution of COVID-19 patients.

The median age of the included patients was 50 years (IQR 35–61 years) (Figure 1). The majority of the patients were between 31 to 60 years (*n* = 686, 57.6%). Only 4.9% (*n* = 59) of the patients were in the age group of 20 years or less. The median age of patients with DM (median—59 years; IQR—50–66 years) was significantly higher than those without DM (median—45 years; IQR—31–57 years) (*p* < 0.001). Gender difference among those with and without DM was not statistically significant (*p* = 0.141).

### 4.2. Comorbidities

Hypertension was the most common comorbidity observed among patients with COVID-19 infection (*n* = 335, 28.1%). CKD was present in 8.1% (*n* = 96), CAD in 6.5% (*n* = 78), cancer in 3.9% (*n* = 46), COPD or bronchial asthma in 2.6% (*n* = 31), and stroke in 1.4% (*n* = 17). Patients with DM had a significantly higher frequency of hypertension, CAD, and CKD (Table 1). There was no difference in the prevalence of stroke, cancer, and COPD/bronchial asthma between the two groups.

The categorical variables are presented as counts and percentages. The continuous variables are presented as median and IQR. The Chi-square test was used for categorical variables and the Mann–Whitney U test for continuous variables. *p* < 0.05 was considered significant. IQR—interquartile range; OR—odds ratio; CI—confidence interval; Y—yes; N—no.

### 4.3. Clinical Features and Presentation

Fever was the most common presentation (*n* = 860, 72.1%), followed by cough (*n* = 484, 40.6%), breathlessness (*n* = 308, 25.8%), and headache (*n* = 99, 8.3%). Ninety percent (1069/1190) of patients presented with a SpO_2_ of 95% or more, 6.2% (74/1190) had a SpO_2_ between 90–94%, and less than 89% was present in 3.9% (47/1190) (data not shown in table). Apart from breathlessness (*p* < 0.001), there was no statistically significant difference in presentation among patients with and without DM. Patients with DM also had a significantly lower SpO_2_ than those without DM (*p* = 0.003). The clinical presentation and basic laboratory data are presented in Table 2.

### 4.4. Investigations

CXR was available for 1118 patients. Normal findings were present in 54.1% (605/1118). In 12.6% (141/1118), there was less than 25% involvement (score 1), and in 33.3% (372/1118) more than 50% of lung fields demonstrated opacities. The median TLC was 6.20 × 10^9^ cells/L (IQR 4.9–8.4 × 10^9^ cells/L). Both TLC and median ANC/ALC were significantly higher in DM.

The categorical variables are presented as counts and percentages. The continuous variables are presented as median and IQR. The Chi-square test was used for categorical variables and the Mann–Whitney U test for continuous variables. *p* < 0.05 was considered significant. OR—odds ratio; CI—confidence interval; CXR—chest X-Ray, score 0—no involvement 0, score 1—less than 25% involvement, score 2—more than 25% involvement 2; TLC—total leucocyte count; ANC/ALC—absolute neutrophil count/absolute lymphocyte count.

### 4.5. Clinical Outcomes in Diabetes

The clinical outcomes in patients with and without DM are summarized in Table 3. The median length of hospital stay for COVID-19 patients was 10 days (IQR—8–14 days). Oxygen was needed in 16.3% (*n* = 193) patients and 13.9% (*n* = 166) were admitted to ICU. An invasive ventilator was required in 6.6% (*n* = 79), non-invasive ventilatory support was provided in 3.2% (*n* = 38), and 2% (*n* = 24) were on both. The median length of ICU stay was 10 days (IQR—5–15.8 days) and ventilation was 5 days (IQR—2–10 days). Diabetes was significantly associated with an increased length of hospitalization (*p* < 0.001), oxygen requirement (*p* < 0.001), ICU admission (*p* < 0.001), and ventilatory requirement (*p* < 0.01).

### 4.6. Mortality

The overall mortality rate in our hospital for patients admitted with COVID-19 infection was 6.1% (*n* = 73). On univariate analysis, the mortality rate was significantly higher in patients with DM (*p* < 0.01, OR 2.3395% CI—1.58–4.12). The other comorbidities that significantly correlated with mortality were hypertension, CAD, stroke, cancer, and CKD. Breathlessness, lower SpO_2_ at presentation, increased infiltrates in CXR, TLC, and ANC/ALC ratio demonstrated significant association with mortality. The relationship between the variables assessed in the study and their relationship to mortality is shown in Table 4.

On multiple logistic regression analysis, only CAD (OR—2.27, 95% CI—1.03–5.02, *p*—0.042), CKD (OR—4.15, 95% CI—1.99–8.64, *p* < 0.001), and cancer (OR—4.29, 95% CI—1.58–11, *p*—0.004) remained significantly associated with mortality, while age, diabetes, hypertension, and stroke did not. The other independent predictors of mortality were breathlessness at presentation, more than 25% involvement in CXR, SpO_2_ at presentation, and ANC/ALC ratio.

## 5. Discussion

This study describes the basic demography, comorbidities, presentation, radiological and laboratory data, and clinical outcome of COVID-19 infected patients with and without DM. Our hospital is a 710 bedded tertiary care center located in an urban setting. The study describes the clinical outcomes of the first 1211 COVID-19 patients admitted to our hospital during the initial phase of the pandemic.

The prevalence of DM in our cohort was 26.8%. It was second only to hypertension, which was the most common (28.1%) comorbidity. A meta-analysis by Sanyaolu et al. [37], involving 1178 patients, reported hypertension in 15.8%, cardiovascular and cerebrovascular diseases in 11.7%, and DM in 9.4%. The meta-analysis by Dorjee K et al. [38] observed a high prevalence of hypertension (55%), DM (33%), smoking history (23%), and heart disease (17%) among hospitalized patients from the USA. A study from north India with 401 patients, reported pre-existing DM in 47.1% and new-onset DM in 5.2% [39]. The prevalence of DM and hypertension in our study is comparable to other reports.

In our study, patients with DM were older than those without DM, but there was no gender difference between the two groups. Other comorbidities such as hypertension, CAD, and CKD were also more common among individuals with DM. Older age and comorbidities were critical predictors of severe disease and mortality in previous reports, and clubbing together of these factors might be interrelated to a worse outcome of COVID-19 infection [39,40,41,42,43].

Fever (72.1%) followed by cough (40.6%), and breathlessness (25.8%) were the most common presentation. Li et al. [44] reported a similar prevalence of fever (88.5%), cough (68.6%), and dyspnea (21.9%). In the meta-analysis by Sun et al. [45], the occurrence of fever (0.891), cough (0.722), and acute respiratory distress syndrome (0.148) were also comparable to our findings. A noteworthy observation was a higher prevalence of breathlessness in patients with diabetes. It is well recognized that diabetes predisposes to more severe disease, and breathlessness at presentation in patients with diabetes might be an early clinical marker for a worse prognosis [46,47].

In our study, patients with DM had more severe involvement in CXR and higher TLC and ANC/ALC ratios. ANC/ALC ratio correlated with severe disease and mortality in previous studies [48,49]. The optimal cut-off value for ANC/ALC ratio in the study by Aly et al. [49] was 3.5. In our study, patients with DM had a median ANC/ALC ratio of 3.5, whereas in patients without DM, the value was 2.47. ANC/ALC ratio has been proposed as an easily available, inexpensive, and reliable prognostic indicator in COVID-19. Lymphopenia is reported in viral infections and severe cases of COVID-19 and results in an elevated neutrophil count [50]. DM was associated with more extensive involvement in CXR in our study. Previous studies have suggested that extensive disease on CXR confers a worse prognosis [51].

DM was found to negatively impact most of the outcomes analyzed in our study. Length of hospital stay, ICU admission, the ventilatory requirement (invasive and non-invasive), and mortality were all adversely influenced by the presence of DM. In several studies and meta-analyses, DM demonstrated consistent association with severe disease and adverse outcomes in COVID-19 infection [38,39,40,41,42,43,44].

The overall mortality rate in our study was 6.1% (71/1192), and among patients with DM, it was 10.7% (34/319). Increased age correlated with higher mortality, though gender did not influence it. Hypertension, CAD, CKD, stroke, and cancer were associated with increased mortality on bivariate analysis but on logistic regression, CKD, cancer, and CAD were the only comorbidities that showed significant association with mortality. In CORONADO, a nationwide multicentric study from France, the variables which independently predicted the primary outcome of tracheal intubation for mechanical ventilation and/or death within 7 days of admission included BMI, dyspnoea, C-reactive protein, and AST [42].

In a meta-analysis of 33 studies by Kumar et al. [12], with 16,003 patients, the pooled prevalence of DM was 9.8% with an odds ratio of 1.90 (*p* < 0.01) for mortality. The presence of DM also correlated with other clinical outcomes such as severe COVID-19, acute respiratory distress syndrome, ICU admission, and disease progression, a finding that has also been replicated in our study [13]. Another meta-analysis of 18 studies with 14,588 patients found an increased risk of mortality in cardiovascular disease, COPD, CKD, cerebrovascular disease, and cancer [24].

The findings of our study are similar to the observations reported in the current literature on COVID-19. However, on logistic regression analysis, DM was not an independent predictor of mortality. In another study from India by Mithal et al., on multiple logistic regression, age, male gender, and baseline severity score were the only independent predictors of mortality. DM and associated comorbidities were significant on univariate analysis but did not reach statistical significance on logistic regression [39]. A study involving institutionalized elderly individuals did not show an association between DM and mortality [52]. In the meta-analysis by Huang et al., studies with a median age > 55 years and prevalence of hypertension > 25% showed a weaker association between DM with adverse outcomes [13]. In our study, though the median age of the entire cohort was 50 years, the median age of patients with DM was 59 years. The overall prevalence of hypertension was 28.1%. Further studies are required to understand, how factors such as age, hypertension, and ethnicity can modify the impact of DM on the outcome of COVID-19 infected patients.

The limitations of our study were its retrospective nature and lack of information related to glycemic status and duration of DM (pre-existing vs. new-onset). We presume that de-novo cases of DM induced by steroid or infection, must have got excluded as self-reported DM or HbA1c during hospitalization would fail to detect and include such cases. There were other variables that influenced the outcome of COVID-19 infection but could not be assessed in this study.

## 6. Conclusions

The presence of DM and related comorbidities exert a significant negative impact on the outcome of COVID-19 infected patients. Older age, hypertension, CAD, CKD, stroke, and cancer were significantly associated with a higher risk of mortality on univariate analysis. On logistic regression analysis, CAD, CKD, and cancer were the only comorbidities that independently predicted mortality. Other indicators of mortality were breathlessness at presentation, extensive involvement in CXR, low SpO_2_ at presentation, and increased ANC/ALC ratio. The presence of these factors could serve as early indicators for severe disease and identify those with a requirement of close monitoring and aggressive treatment.

## Figures and Tables

**Table 1:** Baseline patient characteristics in patients with and without diabetes.

	Number (Percentage)	Diabetes	Non Diabetes	OR (95% CI)	*p* Value
	Total *n* = 1192	Diabetes = 319	Non-Diabetes = 873		
Age (median, IQR)	50 (35–61)	59 (50–66)	45 (31–57)		<0.001
Sex					
Female	360 (30.2)	86 (26.9)	274 (31.3)	1.24 (0.03–1.65)	0.141
Male	832 (69.8)	233 (73.0)	599 (68.6)		
Comorbidities					
Hypertension(Y/N)	335/857 (28.1)	189/146	130/727	7.24 (5.44–9.63)	<0.001
Coronary artery disease(Y/N)	78/1114 (6.5)	43/276	35/838	3.73 (2.34–5.96)	<0.001
Stroke (Y/N)	17/1175 (1.4)	8/311	9/864	2.47 (0.0944–6.46)	0.057
Cancer (Y/N)	46/1146 (3.9)	14/305	32/841	1.21 (0.635–2.29)	0.566
Chronic kidney disease (Y/N)	96/1096 (8.1)	48/271	48/825	3.04 (1.99–4.65)	<0.001
Chronic obstructive pulmonary disease/asthma(Y/N)	31/1161 (2.6)	12/307	19/854	1.76 (0.843–3.66)	0.128

The categorical variables are presented as counts and percentages. The continuous variables are presented as median and IQR. Chi-square test was used for categorical variables and Mann-Whitney U test for continuous variables. *p* < 0.05 was considered significant. IQR—interquartile range, OR—Odds ratio, CI—confidence interval, Y—yes, N—No.

**Table 2:** Clinical, radiological, and laboratory parameters in patients with and without diabetes.

Symptoms	Overall	Diabetes	No Diabetes	OR (95% CI)	*p*-Value
Fever	860/1192 (72.1%)	234/319 (73.4%)	626/873 (71.7%)	1.09 (0.814–1.45)	0.574
Cough	484/1192 (40.6%)	143/319 (44.8%)	341/873 (39.1%)	1.27 (0.978–1.64)	0.073
Breathlessness	308/1192 (25.8%)	108/319 (33.9%)	200/873 (22.9%)	1.72 (1.3–2.28)	<0.001
Headache	99/1192 (8.3%)	21/319 (6.6%)	78/873 (8.9%)	0.718 (0.436–1.18)	0.193
CXR					
Score 0	605/1118 (54.1%)	116/302 (38.4%)	489/816 (59.9%)		<0.001
Score 1	141/1118 (12.61%)	50/302 (16.6%)	91/816 (11.2%)		<0.001
Score 2	372/1118 (33.27%)	136/302 (45%)	236/816 (28.9%)		<0.001
TLC	6.20 (4.9–8.4)	6.6 (5.2–8.9)	6.1 (4.8–8.1)		0.002
ANC/ALC Ratio	2.75 (1.77–4.85)	3.6 (2.23–6.8)	2.47 (1.65–4.13)		<0.001

The categorical variables are presented as counts and percentages. The continuous variables are presented as median and IQR. Chi-square test was used for categorical variables and Mann-Whitney U test for continuous variables. *p* < 0.05 was considered significant. OR—Odds ratio, CI—confidence interval, CXR—chest X Ray, Score 0—no involvement 0, score 1-less than 25% involvement, score 2—more than 25% involvement 2, TLC—total leucocyte count, ANC/ALC—absolute neutrophil count/absolute lymphocyte count.

**Table 3:** Clinical outcome in patients with and without Diabetes.

Outcome	All	Diabetes	No Diabetes	OR (95% CI)	*p* Value
Length of hospital stay(days)	10 (IQR 08–14)	12 (9–16)	10 (8–13)		<0.001
Oxygen requirement	193 (16.2%)	95/319 (29.8%)	98/873 (11.2%)	3.35 (2.44–4.61)	<0.001
ICU Admission	166 (13.9%)	78/319 (24.5%)	88/873 (10.1%)	2.89 (2.06–4.05)	<0.001
Length of ICU Stay	10 (5–16)	10 (5–16)	10 (6–15)		0.611
No Ventilator	1051/1192 (88.2%)	255/319 (79.9%)	796/873 (91.2%)		<0.001
Invasive Ventilator	38/1192 (3.19%)	20/319 (6.27%)	18/873 (2.06%)		
Non Invasive ventilator	79/1192 (6.63%)	37/319 (11.6%)	42/873 (4.81%)		
Both Invasive/Non Invasive	24/1192 (2.01%)	7/319 (2.19%)	17/873 (1.95%)		
Length of Ventilator	5.5 (2–10)	5 (1–12)	7 (3–10)		0.517
Mortality	73/1192 (6.1%)	34/319 (10.7%)	39/873 (4.5%)	2.55 (1.58–4.12)	<0.001

The categorical variables are presented as counts and percentages. The continuous variables are presented as median and IQR. The Chi-square test was used for categorical variables and the Mann–Whitney U test for continuous variables. *p* < 0.05 was considered significant. OR—odds ratio; CI—confidence interval; IQR—interquartile range; ICU—intensive care unit.

**Table 4:** Predictors of mortality in COVID-19 infection.

	Non-Survivors—73	Survivors—1119	Odds Ratio (95% CI)	*p* Value
Age (median, IQR)	62 (53–72)	49 (35–60)		<0.001
Female/Male	18/55	342/777	1.34 (0.78–2.32)	0.287
Comorbidities				
Diabetes (Y/N)	34/39	285/834	2.55 (1.58–4.12)	<0.001
Hypertension (Y/N)	28/45	307/812	1.65 (1.01–2.69)	0.044
CAD (Y/N)	16/57	62/1057	4.79 (2.60–8.81)	<0.001
Stroke (Y/N)	4/69	13/1106	4.93 (1.57–15.5)	0.003
Cancer (Y/N)	9/64	37/1082	4.11 (1.90–8.89)	<0.001
CKD (Y/N)	17/56	79/1040	4.00 (2.22–7.20)	<0.001
COPD/Asthma(Y/N)	3/70	28/1091	1.67 (0.49–5.63)	0.403
Clinical and laboratory indices				
Fever	53/20	807/312	1.02 (0.60–1.74)	0.929
Cough	29/44	455/664	0.962 (0.59–1.56)	0.875
Breathlessness	45/28	263/856	5.23 (3.20–8.55)	<0.001
SpO_2_	95 (90–05)	95 (95–96)		<0.001
Chest X Ray (0/1/2)	12/6/51	593/135/321		<0.001
TLC	8.43 (5.29–12.5)	6.2 (4.87–8.2)		<0.001
ANC/ALC ratio	6.90 (3.42–12.9)	2.64 (1.75–4.48)		<0.001

The categorical variables are presented as counts and percentages. The continuous variables are presented as median and IQR. The Chi-square test was used for categorical variables and the Mann–Whitney U test for continuous variables. *p* < 0.05 was considered significant. OR—odds ratio; CI—confidence interval; IQR—interquartile range; Y—yes, N—no; CAD—coronary artery disease; CKD—chronic kidney disease; COPD—chronic obstructive pulmonary disease; SpO_2_—saturation of peripheral oxygen; TLC—total leucocyte count; ANC/ALC—absolute neutrophil count/absolute lymphocyte count.

## Data Availability

Data is available upon reasonable request to corresponding author.
